# Use of Ozonized Water in the Prevention of Surgical Site Infection in Children Undergoing Cardiovascular Surgery

**DOI:** 10.21470/1678-9741-2023-0006

**Published:** 2023-08-07

**Authors:** Alexandre Noboru Murakami, Ulisses Alexandre Croti, Bruna Cury Borim, Carlos Henrique De Marchi, Rouse Mary Rossini Murakami, Margarete Teresa Gottardo de Almeida, Rafael da Silva Policarpo, Fabiana Nakamura Avona, Moacir Fernandes de Godoy

**Affiliations:** 1 Department of Cardiovascular Surgery, Serviço de Cirurgia Cardíaca do Norte do Paraná, Universidade Estadual de Londrina (UEL), Londrina, Paraná, Brazil; 2 Department of Pediatric Cardiology and Cardiovascular Surgery, CardioPedBrasil® – Hospital da Criança e Maternidade de São José do Rio Preto, São José do Rio Preto, São Paulo, Brazil; 3 Department of Infectious Diseases, Faculdade de Medicina de São José do Rio Preto, São José do Rio Preto, São Paulo, Brazil; 4 Department of Pediatrics, Hospital Evangélico de Londrina, Londrina, Paraná, Brazil

**Keywords:** Ozone, Cross Infection, Congenital Heart Defects

## Abstract

**Introduction:**

Since the reduction of healthcare-associated infections has been a focus for quality patient care, this study aimed to evaluate the surgical site infection rate of children who underwent cardiovascular surgery after implementation of ozonized water system for hand and body hygiene allied to previously implemented preventive measures.

**Methods:**

Two uniformly comparable groups of pediatric patients underwent cardiovascular surgery. Group A (187) patients were operated prior to installation of ozonized water system (March 1 to August 31, 2019), and group B (214) patients were operated after installation of ozonized water system (October 1, 2019, to March 31, 2020). Ozonized water was used for professional hand hygiene and patient body hygiene.

**Results:**

There was statistical significance for surgical site infection reduction in group B (*P*=0.0289), with a relative risk of 0.560 (95% confidence interval = 0.298 to 0.920), inferring the risk of being diagnosed with surgical site infections in group B was 44% less than in group A. There was no statistical significance regarding mechanical ventilation time (*P*=0.1998) or mortality (*P*=0.4457).

**Conclusion:**

Ozonized water for professional hand hygiene and patient body hygiene was an adjuvant combined with traditional preventive methods to reduce the risk of surgical site infection, although no impact on hospital stay or mortality was observed.

## INTRODUCTION

Ozone (O_3_) is an unstable gas and partially soluble in water. It consists of a molecule composed of three oxygen atoms, produced by electrochemical discharge^[1]^. Due to its broad antimicrobial activity with inactivation of microorganisms, it has been used in water disinfection, food industry, dentistry, and medicine^[2,3,4,5]^.

Considering wastewater treatment, its effectiveness has already been demonstrated, showing inactivation of 1 to 2 log units CFU/100 mL of antibiotic-resistant bacteria, and up to 5.5 log units CFU/100 mL of tetracycline-resistant *Escherichia coli*. In addition, O3 has also shown to be effective against chlorine-resistant bacteria^[6,7,8]^. Regarding the most efficient form of O_3_ (if gas or dissolved in water), the use of ozonized water proved to be more viable for decontamination rather than its gas form due to instability and dispersion of gas in the environment. Ozonized water is able to maintain its oxidizing power for a longer time, making it easier and safer to handle than ozonized gas^[9]^.

In order to avoid surgical site infections (SSI), it is recommended to adopt several preventive measures, commonly known as a prevention *bundle*. Some of these activities include hand hygiene, preoperative bath with antiseptic solution, adequate skin preparation before applying antiseptic solution, and surgical wound care, among others^[10,11]^.

This study aimed to evaluate SSI rate of children who underwent cardiovascular surgery after implementation of ozonized water system for hand and body hygiene allied to previously implemented preventive measures.

## METHODS

From March 1, 2019, to March 31, 2020, 401 patients undergoing cardiovascular surgery aged ≤ 18 years were entered into the International Quality Improvement Collaborative for Congenital Heart Disease (IQIC) database of a referral center for treatment of congenital heart disease in Brazil.

Patients were divided into two groups — group A, with 187 patients operated between March 1 and August 31, 2019, period prior to the installation of the ozonized water system, and group B, with 214 patients operated on between October 1, 2019, and Mach 31, 2020, period after the installation of the ozonized water system. The system was installed during the month of September 2019 and, therefore, patients operated that month were excluded from the study.

Both groups A and B underwent the same measures previously implemented for the prevention of SSI (bundle), which include the worldwide technique of hand hygiene and patients’ daily bath with 2% chlorhexidine solution. The difference was that group B patients received all of the pre-established care combined with the ozonized water system.

The previously implemented SSI prevention bundle was divided into three moments, as shown in Table 1.

**Table 1:** Surgical site infection prevention bundle measures implemented before the ozonized water system.

**Prevention measures (preoperative):**
Daily bath with chlorhexidine 2%
Preoperative bath with chlorhexidine 2% (twice)
Oral hygiene with chlorhexidine 0.12%
Trichotomy
**Prevention measures (intraoperative):**
Antibiotic prophylaxis
Operating room hand hygiene antiseptic measures
Degermation and skin preparation
Redosing of the antibiotic during surgical procedure
Operating room laminar flow and air conditioning with proper functioning and parameters
Incision dressing
**Prevention measures (postoperative):**
Antibiotic prophylaxis
Evaluation and maintenance of surgical site dressing
Aseptic technique when performing transthoracic echocardiography

**Fig. 1:**
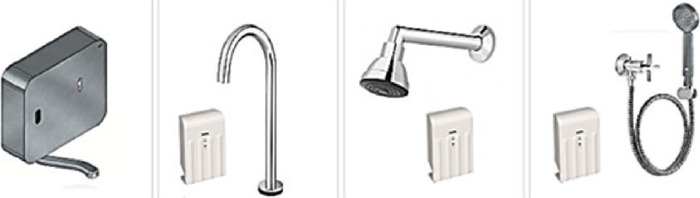
Faucets, shower, and hygienic shower models installed with ozonized water system.

**Fig. 2:**
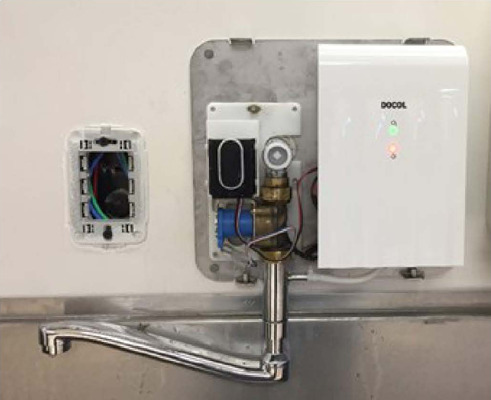
Internal view of ozonized water system faucet.

**Fig. 3:**
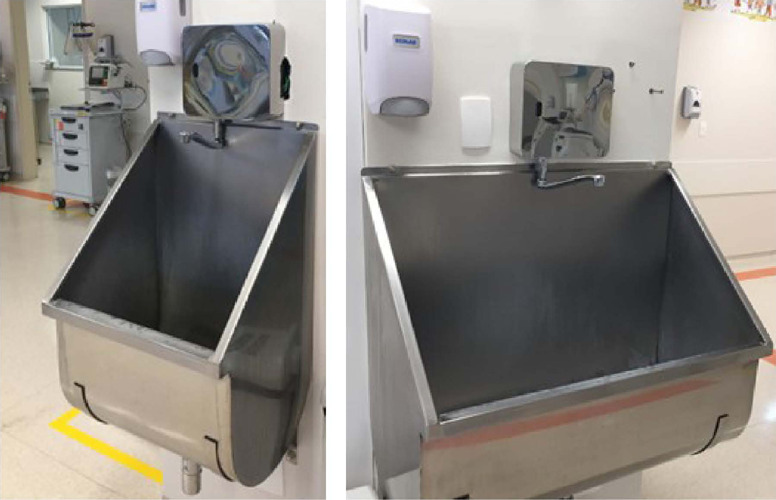
External view of ozonized water system faucet.

A total of 27 DOCOL® brand ozonized water system products (faucets, showers, and hygienic showers) were installed for hand and body hygiene of patients, 25 of them in the intensive care unit (ICU) and two in pediatric cardiovascular surgery operating rooms, as shown in Figures 1, 2, 3.

The ozonized water system used for this study mixes the gaseous O_3_ in the water, produced by an O3 generator coupled to a suction system by an internally located Venturi effect. The electrochemical discharge that synthetically produces the gas known as the corona effect has a production rate of 100 mg/h^[1,12]^. The equipment consists of two electrodes exposed to different action potentials, and the passage of air or pure oxygen between the two electrodes generates O_3_^[2,12]^.

Due to its instability caused by rapid dissociation, it is necessary to generate it on site or as close as possible to the site to be used. After the mixture of gaseous O3 with the water flow, the mixture reaches a dilution in the range of 0.2–0.6 ppm (mg/liter) of O3 in water, according to the manufacturer^[1,13,14]^.

Data collected from electronic medical record were entered and analyzed on the IQIC database platform and included: age at the time of surgery (in months), gender, weight (kg), chromosomal abnormality or associated genetic syndrome, list of procedures by Risk Adjustment for Congenital Heart Surgery (RACHS-1) categories, duration of mechanical ventilation (MV), total length of stay, length of ICU stay, diagnosis and type of SSI, and mortality up to 30 days after surgery or until hospital discharge/death (if discharge/death exceeded 30 days from the operation).

Selected variables included demographic and clinical characteristics of the population and outcome of these patients.

Nominal qualitative variables were compared using the chi-square test or Fisher’s test. For comparison of quantitative variables with Gaussian distribution, the nonparametric *t*-test was used. For comparison of discrete quantitative variables or of continuous quantitative variables without Gaussian distribution, the MannWhitney U test was used.

Diagnosis of SSI and topographic classification were determined according to world standard guidelines and through Hospital Infection Control Service evaluation^[11]^.

An alpha error of 5% was admitted, and *P*-values ≤ 0.05 were considered significant.

The study was approved by the Ethics and Research in Human Beings Committee (Certificado de Apresentação de Apreciação Ética opinion nº 31657920.1.0000.5415). All procedures contributing to this work comply with the ethical standards of the relevant national guidelines on human experimentation (Comissão Nacional de Ética em Pesquisa) and with the Helsinki Declaration of 1975, as revised in 2008, and have been approved by the institutional committees (Comitê de Ética em Pesquisa of the Faculdade de Medicina de São José do Rio Preto [CEP/FAMERP nº 1.541.350]).

The authors had full control of the design of the study, methods used, outcome parameters, analysis of data, and production of the report.

## RESULTS

Groups A and B were uniformly comparable with regard to age, gender, weight, presence of associated genetic syndrome, and RACHS-1 category, as shown in Table 2.

**Table 2:** Comparison between groups A and B patients verifying uniformity among variables.

	Age (months) Mean ± SD Median [range] {Min. - max.}	Gender N (%)	Weight (Kg) Mean ± SD Median [range] {Min. - max.}	Associated genetic syndrome N (%)	RACHS-1 Category: N (%)
**Group B (N = 214)**	21.6±36.2	Male	10.1±11.9	Present	1: 41 (19.2)
	9 [3 – 12]	99 (46.3)	6.9 [4.2 – 11.0]	45 (21)	2: 79 (36.9)
	{0.03 to 228}	Female	{0.8 to 115.6}	Absent	3: 65 (30.4)
		115 (53.7)		169 (79)	4: 29 (13.6)
					5:00
					6:00
**Group A (N = 187)**	24.4±37.1	Male	10.4±9.3	Present	1: 44 (23.5)
	9 [4 – 24]	100 (53.5)	7.2 [4.5 – 12.0]	52 (27.8)	2: 75 (40.1)
	{0.03 to 204}	Female	{1.0 to 47.0}	Absent	3: 50 (26.7)
		87 (46.5)		135 (72.2)	4:13 (7)
					5:00
					6: 5 (2.7)
***P*-value**	0.4668*	0.1798**	0.4188*	0.1430**	0.1067*

RACHS-1=Risk Adjustment for Congenital Heart Surgery; SD=standard deviation

* *P*-value related to the Mann-Whitney U test

** *P*-value relative to the Χ² test

Table 3 shows the number of patients who developed SSI in both groups. There was a statistically significant difference in the occurrence of SSI comparing the months prior to the use of O3 (group A) with the months after installing the ozonized water system (group B), observing a decrease in SSI (*P*=0.0289).

**Table 3:** Number of patients diagnosed with surgical site infection before and after installation of ozonized water system.

Group A			Group B		
	N	Surgical site infection N (%)		N	Surgical site infection N (%)
March 2019	25	2 (8)	October 2019	46	2 (4.3)
April 2019	37	5 (13.5)	November 2019	33	2 (6.1)
May 2019	34	3 (8.8)	December 2019	33	1 (3)
June 2019	26	2 (7.6)	January 2020	37	1 (2.7)
July 2019	23	1 (4.3)	February 2020	40	2 (5)
August 2019	42	5 (11.9)	March 2020	25	0
**Total**	**187**	**18 (9.6)**	**Total**	**214**	**8 (3.7)**

The relative risk was 0.560, with a 95% confidence interval (CI) of 0.298 to 0.920, inferring that the risk of patients being diagnosed with SSI in group B was 44% less than of the patients in group A. Considering the uniformity of both groups, a lower SSI rate in group B suggests a causal relationship, therefore, the use of ozonized water probably was a fundamental and supporting factor in SSI reduction in children undergoing congenital heart surgery.

Regarding length of MV, medians were 23 hours (0.96 days) in group A and 19.2 hours (0.8 days) in group B, so no statistical significance was found between groups A and B (*P*=0.1998).

**Fig. 4:**
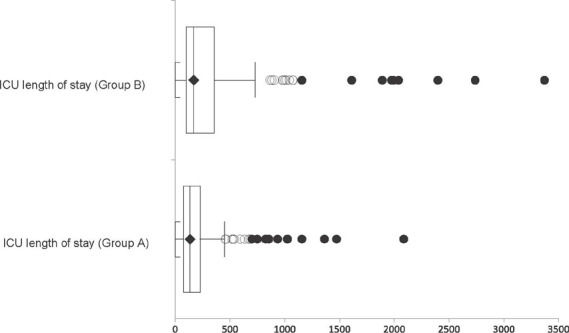
Group comparison of intensive care unit (ICU) length of stay in hours.

A longer ICU stay was found in group B (*P*=0.0002) with a median of 168.7 hours, and 135 hours in group A (7 days *vs.* 5.6 days, respectively), as shown in Figure 4. The median of total length of hospital stay for group A was 11 days and that of group B was 12.5 days (*P*=0.106).

For mortality of patients after 30 days of surgery, there was no statistically significant difference between the groups (*P*=0.4457) and a relative risk of 0.822 (95% CI 0.506 to 1.179). It is worth mentioning that the deaths described are unrelated to SSI diagnosis.

The analysis of specific variables of patients who developed SSI in both groups is described in Table 4. The age groups of patients affected with SSI showed a small difference between ≤ 30 days and > 1 year, with four patients ≤ 30 days of life (22.2%) in group A, one patient (12.5%) in group B, and three patients > 1 year in both groups (16.7% in group A and 37.5% in group B).

**Table 4:** Variables of patients who developed surgical site infection in groups A and B.

		Group A (N=18)	Group B (N=8)
Age range, N (%)	≤ 30 days	4 (22.2)	1 (12.5)
	31 days to 1 year	11 (61.1)	4 (50)
	> 1 year	3 (16.7)	3 (37.5)
Weight, Kg	Mean	8.5	7.5
	Median	6.9	6.9
Genetic syndrome, N (%)		10 (55.6)	4 (50)
Reoperation, N (%)		1 (5.6)	0
RACHS-1, N (%)	Category 1	1 (5.6)	2 (25)
	Category 2	9 (50)	1 (12.5)
	Category 3	6 (33.3)	5 (62.5)
	Categories 4 to 6	2 (11.1)	0
Types of SSI, N (%)	Superficial	16 (88.9)	7 (87.5)
	Deep	2 (11.1)	1 (12.5)
MV time, days	Median	1.4	0.7
ICU LOS, days	Median	9.4	8.4
Total LOS, days	Median	27	22

ICU=intensive care unit; LOS=length of stay; MV=mechanical ventilation; RACHS-1=Risk Adjustment for Congenital Heart Surgery; SSI=surgical site infection

The median weight and presence of genetic syndrome (percentage) were similar in both groups of patients who had SSI, with a median weight of 6.9 kg and presence of an associated genetic syndrome of 55.6% in group A (10 patients) and 50% in group B (4 patients), the most common being trisomy 21 (90 and 75%, respectively) and Di George syndrome (10% and 25%, respectively).

The most common RACHS-1 categories in patients with SSI in group A were category 2 with nine patients (50%) followed by category 3 with six patients (33.3%). In group B, the most common were category 3 with five patients (62.5%) followed by category 1 with two patients (25%). In this second group, there were no patients in categories 4 to 6.

Superficial SSIs were the vast majority in groups A and B, with 16 patients (88.9%) in group A and seven patients (87.5%) in group B, followed by deep SSIs (11.1 and 12.5%, respectively).

Median length of time on MV was 1.4 days in group A and 0.7 days in group B (median 0.96 and 0.8 days, respectively).

The ICU length of stay and total hospital length of stay of patients with SSI were longer when compared to times of the general group of patients. Regarding ICU time, median was 9.4 days in group A (additional 3.8 days to the general sample of this group) and 8.4 days in group B (additional 1.4 days). Regarding the total length of stay, median of group A in infected patients was 27 days (16 days more than the median of the general group), and in group B, the median was 22 days (additional 9.5 days).

## DISCUSSION

Following standardized practices such as prevention bundles facilitates adherence to care by the interdisciplinary team and makes it possible to reduce infection rates of hospitalized patients, including those undergoing pediatric cardiovascular surgery^[15]^.

O3, due to its high oxidizing power, has an antimicrobial activity against filamentous fungi, yeasts, viruses, bacteria, and protozoa, in addition to bacterial and fungal spores, which could become an interesting adjuvant to previously implemented measures to prevent infections in hospital environments^[16,17,18,19]^.

It is important to mention the impact on costs, since infections are a huge concern, especially in developed countries where reimbursements for SSI treatment are being reduced or even denied^[20]^. The ozonized water systems were donated by DOCOL® company for research, without pressuring for positive results, and would total approximately R$17,769.00 (Brazilian reais) or U$3,484.00 (US dollars).

A Brazilian study in 2019 with a pediatric population hospitalized in ICU showed a cost 4.2 times higher in children who developed healthcare-associated infection (HAI), with a median cost of US$10,017.22^[21]^. Therefore, we understand that the cost of installing and using ozonized water is equivalent to less than half of a case of HAI, and that the reduction of SSI by 44% in six months of using ozonized water provides an exponential reduction of hospital costs. Reducing infections is even more complex when dealing with children undergoing pediatric cardiovascular surgery, as these patients are somehow debilitated, either by a deficiency or associated genetic syndrome, or by some preoperative condition that may cause complications during the postoperative period^[22]^. Approximately 50% of the patients affected by SSI in both groups had some genetic syndrome, trisomy 21 being the most common. These patients are more susceptible to viral and bacterial infections, and this has been attributed to the compromise of the immune system, one of the pathological features of trisomy 21^[23,24]^.

Nutritional status in congenital heart disease patients is dependent on the type and hemodynamic repercussion of the defect. The greater the hemodynamic compromise, the greater the difficulties encountered in maintaining the expected pattern of growth and development^[25]^. Change in preoperative nutritional status can also affect wound healing, which depends on deficient serum proteins in malnourished patients^[26]^. There was similarity in patient weight of both group samples, demonstrating uniformity between them. The RACHS-1 classification serves as a reference for the severity of surgical correction, estimating the potential for postoperative complications such as the incidence of SSI in postoperative period of pediatric cardiovascular surgery^[27]^.

Patients in both groups were comparable in relation to RACHS-1 (*P*=0.1067) and, interestingly, among patients who developed an SSI, the percentages were lower for categories 4 to 6 (11.1% in group A and 0% in group B). Therefore, we could infer more complex patients would better benefit from the use of the ozonized water system to improve results and lower hospital costs. SSI is considered the most impacting postoperative complication because affected patients are two to 11 times more susceptible to death, longer hospital stay, and, consequently, higher hospital costs^[28,29]^. There was no statistically significant difference between groups regarding risk of death (*P*=0.4457), however, there was clinical relevance due to the loss of three more patients in group A (15 patients) than in group B (12 patients).

There was no statistical significance in MV time (*P*=0.1998) probably due to a few outliers in group B, as shown in Figure 4. Regarding ICU length of stay, there was no important clinical relevance for group B when transforming hours into days (7 days for group B *versus* 5.6 days for group A).

### Limitations

As for study limitations, due to the coronavirus disease 2019 (or COVID-19) pandemic taking place in our region in mid-March 2020, a pause in data collection was necessary since the number of patients operated was lower than expected, patient profile changed due to reduction in elective surgeries, and critically ill newborns previously hospitalized in other services in use of antibiotic therapy were transferred to our facility. In addition, hand hygiene was intensified, along with use of masks, which could have an impact on infection reduction.

## CONCLUSION

As a hypothesis to be studied in the future, the impact on hospitalization time, costs, and mortality of patients diagnosed with SSI with the complete use of ozonized water (as a preventive measure and treatment option) can be evaluated.

No samples of microorganisms identified in the SSIs were collected, so there is another potential future study on the effectiveness of ozonized water in the treatment of specific organisms found in infected/colonized surgical wounds.

Despite the complexity of patients, nutritional status, and organic repercussions related to the procedure, both uniformly separated groups showed statistically significant differences in SSI rates after implementation of hand and body hygiene technique with the ozonized water system.

Therefore, we infer that ozonized water for professional hand hygiene and patient body hygiene was an adjuvant combined with traditional preventive methods to reduce the risk of SSI, although no impact on hospital stay or mortality was observed.

## Abbreviations, Acronyms & Symbols

CI: Confidence interval
HAI: Healthcare-associated infection
ICU: Intensive care unit
IQCI: International Quality Improvement Collaborative for Congenital Heart Disease
LOS: Length of stay
MV: Mechanical ventilation
O_3_: Ozone
RACHS-1: Risk Adjustment for Congenital Heart Surgery
SD: Standard deviation
SSI: Surgical site infections
